# Amyloid Precursor Protein Translation Is Regulated by a 3’UTR Guanine Quadruplex

**DOI:** 10.1371/journal.pone.0143160

**Published:** 2015-11-30

**Authors:** Ezekiel Crenshaw, Brian P. Leung, Chun Kit Kwok, Michal Sharoni, Kalee Olson, Neeraj P. Sebastian, Sara Ansaloni, Reinhard Schweitzer-Stenner, Michael R. Akins, Philip C. Bevilacqua, Aleister J. Saunders

**Affiliations:** 1 Department of Biology, Drexel University, Philadelphia, PA, United States of America; 2 Department of Chemistry, Drexel University, Philadelphia, PA, United States of America; 3 Department of Chemistry, Pennsylvania State University, University Park, PA, United States of America; 4 Department of Biochemistry & Molecular Biology, Center for RNA Molecular Biology, Pennsylvania State University, University Park, PA, United States of America; 5 Department of Chemistry, University of Cambridge, Cambridge, United Kingdom; 6 Department of Biochemistry & Molecular Biology, Drexel University College of Medicine, Philadelphia, PA, United States of America; 7 Department of Neurobiology & Anatomy, Drexel University College of Medicine, Philadelphia, PA, United States of America; Sungkyunkwan University, REPUBLIC OF KOREA

## Abstract

A central event in Alzheimer’s disease is the accumulation of amyloid β (Aβ) peptides generated by the proteolytic cleavage of the amyloid precursor protein (APP). APP overexpression leads to increased Aβ generation and Alzheimer’s disease in humans and altered neuronal migration and increased long term depression in mice. Conversely, reduction of APP expression results in decreased Aβ levels in mice as well as impaired learning and memory and decreased numbers of dendritic spines. Together these findings indicate that therapeutic interventions that aim to restore APP and Aβ levels must do so within an ideal range. To better understand the effects of modulating APP levels, we explored the mechanisms regulating APP expression focusing on post-transcriptional regulation. Such regulation can be mediated by RNA regulatory elements such as guanine quadruplexes (G-quadruplexes), non-canonical structured RNA motifs that affect RNA stability and translation. Via a bioinformatics approach, we identified a candidate G-quadruplex within the APP mRNA in its 3’UTR (untranslated region) at residues 3008–3027 (NM_201414.2). This sequence exhibited characteristics of a parallel G-quadruplex structure as revealed by circular dichroism spectrophotometry. Further, as with other G-quadruplexes, the formation of this structure was dependent on the presence of potassium ions. This G-quadruplex has no apparent role in regulating transcription or mRNA stability as wild type and mutant constructs exhibited equivalent mRNA levels as determined by real time PCR. Instead, we demonstrate that this G-quadruplex negatively regulates APP protein expression using dual luciferase reporter and Western blot analysis. Taken together, our studies reveal post-transcriptional regulation by a 3’UTR G-quadruplex as a novel mechanism regulating APP expression.

## Introduction

Amyloid plaques and neurofibrillary tangles are characteristic pathologic features of Alzheimer’s disease (AD), a progressive neurodegenerative disorder and the most common form of dementia [1]. Amyloid plaques are formed from the amyloid β peptide (Aβ), which is a proteolytic product of the amyloid precursor protein (APP). APP is a type 1 transmembrane protein that is ubiquitously expressed in humans [2]. While the biological function of APP remains obscure, a large body of work indicates that APP plays a critical role in AD pathogenesis via production of Aβ [2]. APP undergoes regulated intramembrane proteolysis (RIP) by one of two proteases, α- or β-secretase. Cleavage by α-secretase in the non-amyloidogenic pathway releases a secreted APP fragment (s-APP α) as well as a transmembrane α C-Terminal Fragment (CTF). Cleavage by β-secretase in the amyloidogenic pathway produces s-APP β and β CTF (for review see [3–7]). APP CTFs can be further cleaved by β-secretase to produce p3 and APP Intracellular domain (AICD) in the non-amyloidogenic pathway or Aβ and AICD in the amyloidogenic pathway [5, 7–9]. Aβ peptides can accumulate and form oligomers that eventually give rise to amyloid plaques [10]. The accumulation of Aβ oligomers can lead to synaptic loss and neurodegeneration [11]. Rare, early-onset forms of AD arise from mutations leading to elevated Aβ production. This change in Aβ can arise from heightened APP levels due to mutations in *APP* or from increased APP copy number as observed in Down’s syndrome (Trisomy 21) [12, 13]. Early onset AD can also arise from elevated Aβ levels due to altered APP processing caused by mutations in the γ-secretase genes *PSEN1* or *PSEN2* [2].The accumulation of Aβ peptides is thought to lead to tau hyperphosphorylation, which can result in synaptic dysfunction, neuronal death, and cognitive decline [14]. Elevated APP expression, and the associated increase in Aβ production via the amyloidogenic pathway, therefore has deleterious effects on both neuronal and cognitive function.

Decreased levels of APP also lead to pathological changes in the brain, as revealed by studies investigating genetically modified mice that lack APP. Acute knock down of APP in neuronal precursor cells prevents these cells from migrating into the cortical plate [15]. Additionally, mice lacking APP exhibit defects in synapse formation that manifest as decreased dendritic spine abundance [16]. The synapses that do form exhibit altered plasticity, as they have impaired long term potentiation [16–18]. Therefore, as with overexpression of APP, reduced levels of APP leads to negative changes in neuronal structure and function.

As both over- and under-expression of APP can be deleterious, identifying the endogenous mechanisms that normally maintain APP expression within the physiological range is of particular interest. Regulatory sequences within the 5’ and 3’ UTRs (untranslated regions) of an mRNA can affect its stability, transcription, and translation and therefore contribute to the spatial and temporal regulation of gene expression [19–23]. One mechanism whereby regulatory RNA sequences alter translation is through RNA secondary structures [24–26]. Guanine quadruplexes (G-quadruplexes), one such secondary structure [27–29], are DNA or RNA sequences containing repeating guanines arranged in a manner that facilitates intra-molecular assembly of stacks of guanine tetrads [30]. Stacking of these guanine tetrads is stabilized by monovalent cations, especially K^+^ and Na^+^ ions [28]. G-quadruplexes require two or more stacks of guanine tetrads [31]. Both DNA and RNA G-quadruplexes form in cells [32, 33], although RNA G-quadruplexes are more stable than DNA G-quadruplexes [34].

Here we investigated the endogenous mechanisms that regulate APP expression. The APP 3’UTR contains a variety of regulatory sequences that affect the stability and ultimately translation of the APP mRNA [35]. We show that a G-rich region in the 3’ UTR of *APP* is a G-quadruplex. Further, we demonstrate for the first time that this sequence negatively regulates APP gene expression in a post-transcriptional manner. These findings are consistent with previous reports demonstrating that 3’UTR G-quadruplexes can negatively regulate the expression of genes that harbor such structures. Moreover, our results suggest this secondary structure is a novel mechanism regulating APP gene expression and therefore may be an important factor contributing to AD pathogenesis.

## Materials and Methods

### APP G-quadruplex identification and sequence conservation

Using Quadparser, the sequence for APP mRNA (NM_201414.2) was searched for putative G-quadruplex sequences following the sequence motif (G_≥ 2_N_1–7_) _3_G_≥ 2_ which defines four repeats of at least two guanines (G) interrupted by stretches of one to seven nucleotides of any type (N) [36]. Alignment for APP 3’UTR G-quadruplex sequence was performed using QGRS-H Predictor software [37] using GeneBank Accession numbers as indicated in the text for APP of different species. Potential G-quadruplexes were analyzed approximately 718 nucleotides downstream from the stop codon.

### RNA preparation

RNA oligonucleotides (APP 3’UTR G-quad Wild type: 5’-GGGGCGGGUGGGGAGGGG-3’) and (APP 3’UTR G-quad Mutant: 5’-GGGGCGGGUGGGGAAAAA-3’) were purchased from Dharmacon, Inc. and Intergrated DNA Technologies. Li^+^ ions prevent Na^+^- or K^+^-induced G-quadruplex formation. To ensure that G-quadruplexes did not form in the analyzed oligonucleotides prior to conducting experiments, the RNA was stored in buffers containing Li^+^ ions. To replace cations in the RNA solution with Li^+^, RNA was dialyzed as described previously [38] in an eight-well microdialysis apparatus (Gibco-BRL Life Technologies) at a flow rate of 25 mL/min. The RNA was initially dialyzed with 100 mM LiCl for 6 h to replace the RNA backbone cation, followed by nuclease-free water for another 6 h to remove excess LiCl, and finally with 10 mM LiCacodylate (pH 7.0) overnight. Concentrations of the dialyzed RNA were quantified by UV-spectroscopy, and the RNA was stored at –20°C until the experiments.

### Circular Dichroism (CD) Spectroscopy

CD spectroscopy experiments were conducted based on a previously published protocol [38] using a Jasco CD J810 Spectropolarimeter and analyzed with KaleidaGraph v.4.5.2 (Synergy Software). RNA oligonucleotides were prepared to a concentration of 2.5 μM in 10 mM LiCacodylate (pH 7.0) by the dialysis procedure described above. Prior to CD, RNAs were denatured at 95°C for 2 min and renatured at room temperature for 15 min for equilibration. Spectra were acquired every nanometer from 220–310 nm at 25°C. Each reported spectrum is an average of 2 scans with a response time of 2 s/nm. Data were normalized to concentration, oligonucleotide length and cuvette pathway to provide molar residue ellipticity values and smoothed over 5 nm [39].

### Data fitting

CD-detected titrations were performed with KCl to determine the concentration of potassium ion (K^+^) required to drive G-quadruplex formation. To determine K^+^_1/2_ values, ellipticity data as a function of K^+^ concentration were fit with KaleidaGraph v. 4.5.2 (Synergy software) according to the apparent three-state Hill equation as previously described [38].

$$\varepsilon =\frac{\varepsilon_{\text{U}}+\varepsilon_{\text{I}}{\left(\frac{{[\text{K}}^{+}]}{{{[\text{K}}^{+}{}_{1/2}]}_{1}}\right)}^{n_{1}}+\varepsilon_{\text{F}}{\left(\frac{{[\text{K}}^{+}]}{{{[\text{K}}^{+}{}_{1/2}]}_{1}}\right)}^{n_{1}}{\left(\frac{{[\text{K}}^{+}]}{{{[\text{K}}^{+}{}_{1/2}]}_{2}}\right)}^{n2}}{1+{\left(\frac{{[\text{K}}^{+}]}{{{[\text{K}}^{+}{}_{1/2}]}_{1}}\right)}^{n_{1}}+{\left(\frac{{[\text{K}}^{+}]}{{{[\text{K}}^{+}{}_{1/2}]}_{1}}\right)}^{n_{1}}{\left(\frac{{[\text{K}}^{+}]}{{{[\text{K}}^{+}{}_{1/2}]}_{2}}\right)}^{n2}}$$ (Eq 1)

where *ε* is the normalized molar ellipticity, *ε*_U_ is the normalized CD signal for fully unfolded RNA, *ε*_I_ is the normalized CD signal for intermediate state RNA, and *ε*_F_ is the normalized CD signal for fully folded RNA. [K^+^_1/2_]_1_ and n_1_ are the K^+^_1/2_ and Hill coefficient values for the U-to-I transition, while [K^+^_1/2_]_2_ and n_2_ are the values for the I-to-F transition. Data were collected at the maximum wavelength (λ_max_) in the spectrum.

### Constructs and Site-Directed-Mutagenesis

The base vector for the luciferase experiments was pMIR-Report miRNA expression reporter vector which contains the firefly luciferase gene (Promega). To create the wild type (WT) construct, the entire 1.1 kb sequence of the APP 3’UTR (NM_201414.2) was placed 3’ of the Luciferase gene stop codon. To create a 3’UTR mutant construct, site-directed mutagenesis (Stratagene, Quikchange mutagenesis kit) was carried out on the wild-type 3’UTR construct to mutate the final four guanine nucleotides of the G-quadruplex to adenine nucleotides (5’-CCCTGTTCATTGTAAGCACTTTTACGGGGCGGGTGGGGAAAAATGCTGGTCTTCAATTAC-3’ and 5’-GTAATTGAAGACCAGCATTTTTCCCCACCCGCCCCGTAAAAGTGCTTACAATGAACAGGG-3’) [38]. Mutagenesis was confirmed by DNA sequencing (Macrogen).

In order to create the human APP-APP 3’UTR over-expression plasmid, we amplified the coding region of human APP and subcloned into the pmCherry-N1 vector using the NheI (5’-CGACGACGAGCTAGC ATGCTGCCCGGTTTGGCA-3’) and XmaI sites (5’-TCGTCGTCGCCCGGGCGTTCTGCATCTGCTCAAAGAA-3’). The human wild-type 3’UTR of APP was amplified and subcloned 3’ of the APP stop codon using the AgeI site underlined (5’-CGACGAACCGGTACCCCCGCCACAGCAGC-3’) sense and (5’-CGGCGGCGGACCGGTGCTCCTCCAAGAATGTATTTATTTAC-3’) antisense. The resulting plasmid was then subjected to site-directed mutagenesis (QuikChange; Stratagene) to change the final four guanine nucleotides of the G-quadruplex to adenine nucleotides [40]. Mutagenesis was confirmed by DNA sequencing.

Using these APP overexpression constructs bearing either the wild type or mutant 3’UTR G-quadruplex, we inserted a c-Myc epitope tag before the APP_695_ stop codon using site-directed mutagenesis with mutagenic primers (5’gagcagatgcagaacgaacaaaaacttatttctgaagaagatctgtagcccgggatccac 3’ and 5’gtggatcccgggctacagatcttcttcagaaataagtttttgttcgttctgcatctgctc 3’.) The underlined nucleotide sequence corresponding to the c-Myc amino acid sequence “EQKLISEEDL.”

### Cell Culture and Transfection

Experiments were carried out in HeLa and HEK293 cells (as indicated in the Results), purchased from the American Type Culture Collection (ATCC). Cells were cultured in Dulbecco’s modified Eagles medium (DMEM), supplemented with 10% fetal bovine serum, L-glutamine (2%), penicillin (25,000 U/ml) and streptomycin (25,000 μg/ml). One day before transfection 4x10^5^ naïve HeLa or HEK293 cells were seeded in 6 well plates. The next day, the medium was replaced with a transfection medium containing the plasmids and Turbofect (ThermoScientific) according the manufacturer’s instructions.

For the metabolic labeling experiments, HeLa cells were transfected using Lipofectamine 3000 (ThermoScientific) according to the manufacturer’s recommendations. Briefly, the HeLa cells were cultured in complete Dulbecco’s modified Eagles medium (DMEM) as described above. One day before transfection, HeLa cells were seeded in 10 cm tissue culture dishes such that they would reach 90% confluence the following day. On the next day, medium was aspirated and replaced with antibiotic free medium containing 10% FBS and 2%L-glutamine. APP-Myc constructs containing either wild type or mutant 3’UTR G-quadruplex sequence were transfected. 6 hours post transfection, transfection medium was aspirated and replaced with complete medium.

### Metabolic Labeling using L-azidohomoalaine (AHA)

24 hours post transfection, cells in 10 cm dishes were prepared for metabolic labeling to examine newly synthesized proteins. Cells were washed twice with warm PBS. DMEM containing only high glucose was added to cells to deplete endogenous methionine and cysteine for 45 minutes. Next, the medium was replaced with DMEM containing 10% dialyzed FBS, 2% L-glutamine, .2 mM L-cysteine, 25 mM HEPES, 1 mM sodium pyruvate, penicillin (25,000 U/ml) and streptomycin (25,000 μg/ml), and 4 mM L-azidohomoalanine (Click Chemistry Tools) for 4 hours. After this incubation period, cell lysates were collect in RIPA buffer and prepared for immunoprecipitation.

### Immunoprecipitation

Following metabolic labeling of cells, lysates were prepared for immunoprecipitation using Dynabeads magnetic Protein G Beads (Life Technologies) following the manufacturer’s instructions. The antibody used for immunoprecipitation was 9B11 C-Myc antibody (Cell Signal) to capture APP_695_Myc (wild type or mutant G-quadruplex) reporter constructs but not the endogenous APP. Following elution, 40% of the eluate along with the immunoprecipitation input and flow through were used for Western Blot Analysis to detect APP using the c1/6.1 mouse monoclonal antibody. The remainder of the eluate was used for the “Click Chemistry” reaction.

### Click Chemistry Reaction

To perform this reaction, IP eluate was subjected to the Click Chemistry Protein Reaction Kit (Click Chemistry Tools). In this reaction a desthiobiotin molecule containing an alkyne group forms a covalent bond to proteins containing the AHA labels (via the AHA’s azide group). This reaction followed the manufacturer’s instructions with the exception that the 30 min incubation time was changed to 1 hour. Following the reaction, samples were prepared for Western blot.

### Western Blot

24 hours post transfection, cells were lysed in RIPA cell lysis buffer (50 mM Tris-HCl pH 7.4, 150 mM NaCl, 1 mM EDTA, 1% NP-40) containing 1X protease inhibitor cocktail (Thermo Scientific). Lysates were centrifuged at 14,000 rpm for 15 minutes at 4°C. The resulting supernatants were transferred to a new microcentrifuge tube and the cell lysate protein concentration was determined using the BCA protein assay kit (Pierce) according to the manufacturer's instructions.

Equal quantities of protein (~40 μg) were mixed with loading buffer and loaded into the wells of 4–12% Bis-Tris polyacrylamide gels (Invitrogen) along with molecular weight standard (LiCor). Gels were run using MES running buffer and transferred to PVDF membrane (Immobilon PSQ, Millipore) using a semi-dry transfer apparatus (Owl Scientific) and NuPage transfer buffer (Invitrogen). After transfer, membranes were blocked with Odyssey Blocking Buffer (LiCor) for 1.5 hours. Next, the blocking buffer was removed and the membrane probed overnight at 4°C with blocking buffer containing C1/6.1 C-Terminal antibody (a gift from Paul Matthews;, 1:4000) and an antibody to β-actin (Sigma; 1:10,000) which were diluted in blocking buffer. Membranes were washed with 1X PBST for 5 minutes (4 times). After the washes, the membrane was probed for 1 hour at room temperature with goat anti-mouse 2° Antibody (800 nm; LiCor) at 1:10,000 diluted in blocking buffer. For the metabolic labeling studies, IRdye 800-conjugated Streptavidin (1:10,000; Licor) was used to detect proteins containing desthiobiotin. Membranes were washed extensively and then scanned using the LiCor Odyssey system. Band intensities were quantified using the Odyssey software.

### Luciferase Assays

Cells were split the day prior to transfection and plated to 40–50% confluency in either 24- or 96-well plates. On the day of transfection, media was aspirated and replaced with fresh media and plasmids transfected using Turbofect (ThermoScientific) according to the manufacturer’s instructions. An excess of pMIR-Luciferase plasmid was transfected relative to the transfection control plasmid (pRL-TK Renilla luciferase); a 40 to 1 molar ratio was utilized. 24 hours post-transfection, media was removed and Glo Lysis Buffer (Promega) was added to each well to lyse cells. These lysates were frozen (-80°C) prior to performing Dual Glo Luciferase Assay (Promega). Firefly Luciferase and pRL-TK Renilla luciferase activity were measured per the manufacturer’s instructions. In all cases, firefly luciferase values were normalized to Renilla luciferase values.

### RNA isolation and Real Time PCR

To collect the lysates for RNA isolation, media from the 6-well plates was aspirated and replaced with 600 μL of RLP buffer (Qiagen, RNeasy Mini Kit) and 60 μL of β-mercaptoethanol (Sigma). All equipment used in the RNA isolation procedure was cleaned with RNaseZap (Ambion) solution. Total RNA was DNase treated by RQ1 RNase-free DNase (Promega), and the RNA concentration was determined spectrometrically (Nano-Drop; Thermo Scientific). cDNA synthesis from total RNA utilized random primers (Invitrogen), Super Script II (Invitrogen), RNase H (Invitrogen), dNTPs (Invitrogen), and RQ1 (Promega). Synthesized cDNA was utilized for real time PCR. We designed primers and probes for firefly and renilla luciferase (Firefly Luciferase primers- 5’-GCTATTCTGATTACACCCGAGG-3’, 5’-TCCTCTGACACATAATTCGCC-3’, 5’-6-FAM-TCCAGATCCACAACCTTCGCTTCAAA-TAMRA-3’; Renilla Luciferase primers- 5’-CAAAGAGAAAGGTGAAGTTCGTC-3’, 5’-gtggtaaacctgacgttgtac-3’, 5’-FAM-atcatggcctcgtgaaatcccgt-TAMRA-3’) and used these in combination with a BioRad 384 well real time thermocycler (CFX384). Taqman Universal PCR Master Mix was used following the manufacturer’s cycling conditions.

### Aβ ELISA

Aβ_40_ ELISA (Wako) was performed using conditioned media from cells transfected with APP_695_ overexpression plasmids containing either the wild type or mutant G-quadruplex sequence following the manufacturer’s instructions.

### Statistical Analysis

All experiments were repeated 3 times unless stated otherwise. All errors are shown as standard error of the mean. Equal variance was assumed for the two-sample student’s t-test (95% confidence interval). Q test was used to reject outliers at the 95% confidence interval. * indicates a P < 0.05, ** indicates a P < 0.01, and *** indicates a P < 0.001.

## Results

### Bioinformatic identification and sequence conservation of an APP 3’UTR G-quadruplex

**Fig 1:**
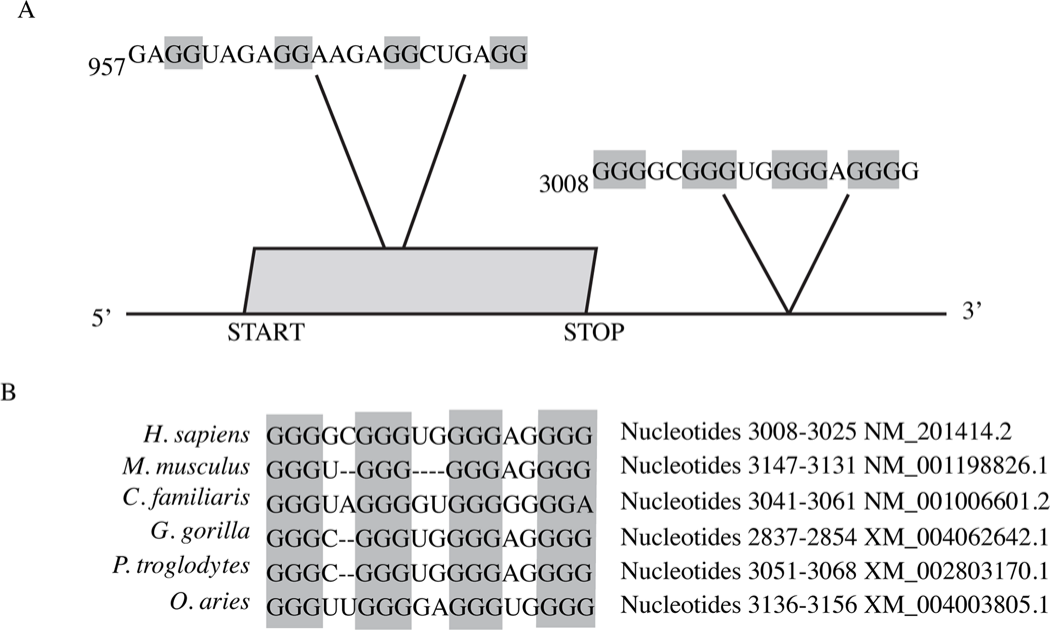
APP contains a putative G-quadruplex. (A) Schematic of the APP mRNA that contains the putative G-quadruplex sequence in the coding region at position 957 (as identified by Westmark *et al*. 2007) and the G-quadruplex sequence in the 3’UTR (discussed in this paper) at position 3008. (B) G-quadruplex consensus sequence and comparison of the G-quadruplex sequence in the 3’UTR of human APP with the 3’UTR APP for other species.

To investigate the post-transcriptional regulation of APP expression, we asked whether the human *APP* mRNA contains a G-quadruplex. Using Quadparser [36], we searched the APP mRNA sequence (NM_201414.2) for putative G-quadruplex sequences following the sequence motif, (G_≥ 2_N_1–7_) _3_G_≥ 2_ which defines four repeats of at least two guanines (G) interrupted by stretches of one to seven nucleotides of any type (N). This approach predicted two putative G-quadruplexes within APP mRNA (Fig 1A). One such sequence is located within the protein coding region beginning at nucleotide 957, consistent with earlier findings [29]. This potential G-quadruplex is predicted to be relatively weak since it has the potential to form a quadruplex with only two stacks of guanine tetrads and the intervening loops are relatively long with 4 nucleotides each [41]. A second putative G-quadruplex was identified within the 3’UTR beginning at nucleotide 3008. This 3’UTR sequence was recently identified independently by a bioinformatic analysis but was not experimentally investigated [42]. This putative G-quadruplex could form a relatively strong G-quadruplex since it has the potential to form a quadruplex comprised of three guanine tetrads and the intervening loops are only 2 or 1 nucleotides in length [31]. Since the G-quadruplex found in APP 3’UTR is approximately 718 nucleotides from the stop codon, we searched the APP gene of several species for potential G-quadruplexes around the same nucleotide distance from the stop codon and was able to obtain an alignment for the comparison of the genes using GQRS-H Predictor software [37] (Fig 1B). The potential functional importance of this 3’UTR G-quadruplex is highlighted by its conservation in *APP* genes in various species (Fig 1B). These findings suggest the presence of functional G-quadruplexes in the *APP* mRNA.

### Structural Confirmation of the APP 3’UTR Putative G-quadruplex

**Fig 2:**
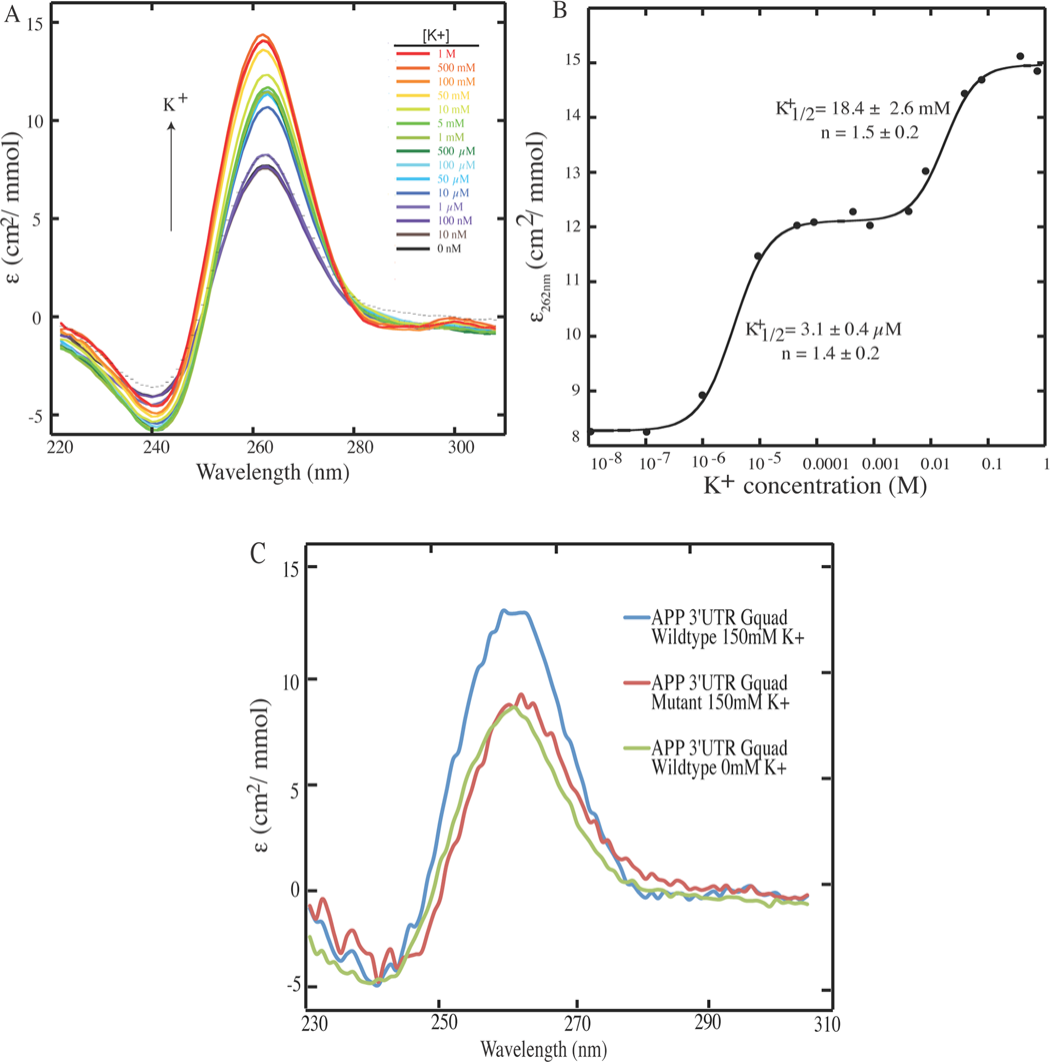
CD potassium ion titration of the *APP* 3’UTR G-quadruplex shows that it forms *in vitro* and is in parallel topology with 3-state folding. (A) CD spectra collected as a function of K^+^ ion concentration. K^+^-mediated G-quadruplex folding is performed at 2.5 μM RNA under 10 mM lithium cacodylate (LiCac) (pH 7.0), with K^+^ ion concentration ranged from 0 to 1 M. The positive peak at ~260 nm and negative peak at ~240 nm are CD signatures for parallel topology of G-quadruplex. (B) CD signal (ellipticity monitored at 262 nm) as a function of K^+^ ion concentration from panel A shows clear three-state transitions in G-quadruplex folding. The fitting was performed using Eq 1 (see Material and Methods). At physiological K^+^ ion concentration (~150 mM), the G-quadruplex is fully folded. The K^+^_1/2_ and Hill coefficients (n) are provided in the plot. (C) CD titration and comparison of APP 3’UTR wild-type and mutant G-quadruplex sequence. 2.5 μM RNAs were used under 10 mM LiCac (pH 7.0) and physiological 150 mM or 0 mM K^+^ ion concentration The GGGG to AAAA substitution in the mutant disfavor G-quadruplex formation as evident by the reduction in CD characteristic signals for G-quadruplex (compare blue and red), and yield similar CD signal to the wild type sequence at 0 mM K^+^ ion concentration (green).

To validate that the sequence identified by the bioinformatic approach is a bona fide G-quadruplex, we performed a structural characterization of this sequence. Several factors contribute to the folding of an RNA into a G-quadruplex, including the sequence itself (guanine tracts, loop sequence, and loop length) as well as the cellular environment (pH, temperature, and the concentration and identities of monovalent cations) [31, 43–45]. Importantly, potassium (K^+^) ions preferentially stabilizes G-quadruplex structures in comparison to sodium (Na^+^) and lithium (Li^+^) ions [46]. G-quadruplex formation can be monitored through key spectral signatures using circular dichroism (CD) spectroscopy. To test whether the 3’UTR sequence forms a G-quadruplex, we used an RNA oligonucleotide bearing the putative APP 3’UTR G-quadruplex and performed K^+^ ion titration monitored by CD. The first immediate observation is that CD spectra of this RNA includes a negative peak at 240 nm and a positive peak at 262 nm (Fig 2A), which are the distinctive CD signatures for a parallel G-quadruplex structure [38, 47]. We next investigated the K^+^ ion dependence of this G-quadruplex structure. Plotting the change in ellipticity versus K^+^ ion concentration revealed a three-state transition (Fig 2B), with a K^+^_1/2_ of ~3 μM and ~18 mM. As the physiological K^+^ concentration is ~150 mM, this result suggests that the APP 3’UTR G-quadruplex is fully folded *in vivo*, with a maximum of 3-quartet planes. We then used CD to compare the oligonucleotide representing the wild-type APP 3’UTR G-quadruplex to the spectrum of the oligonucleotide representing a APP 3’UTR G-quadruplex sequence in which the fourth set of G repeats was replaced with adenines (APP 3’UTR G-Quad Mutant) [40, 48]. This was conducted in the presence of 150 mM KCl, which induces G-quadruplex formation in the wild-type sequence. In this mutated oligonucleotide we observed a significant decrease in the 262 nm peak, indicating a decrease in population of the G-quadruplex fold (Fig 2C). Taken together, our CD data support the presence of a parallel G-quadruplex that is stabilized by K^+^ ions.

### The APP 3’UTR G-quadruplex does not regulate expression levels of the APP RNA

**Fig 3:**
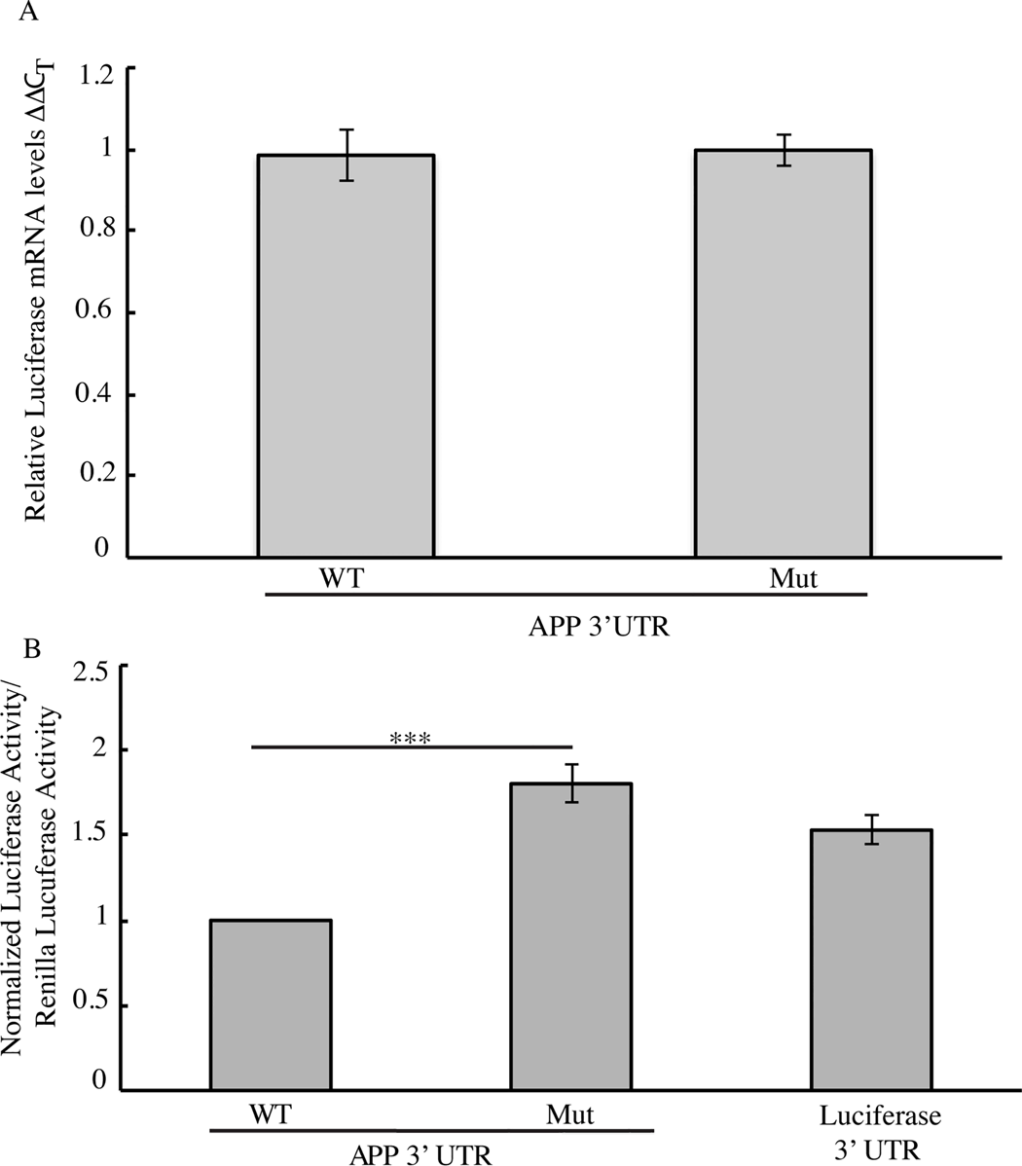
G-quadruplex regulation of Luciferase gene expression. (A) mRNA levels as assessed by qPCR and represented ΔΔC_T_ values represents the normalization of the ΔC_T_ of firefly luciferase to the ΔC_T_ renilla luciferase then normalized to the wild type. (B) Quantification of Dual Luciferase Assay comparing the wild type G-quadruplex sequence to the mutant G-quadruplex sequence (G-quad Mut). Empty Firefly Luciferase plasmid was used as a control which does not contain the 3’UTR of APP. Firefly Luciferase values were normalized to Renilla Luciferase.

We next investigated the functional significance of this G-quadruplex. As these structures can play roles in transcription, RNA stability and translation[28], we wanted to investigate what role the APP 3’UTR G-quadruplex has on gene expression. We began these studies by investigating whether the G-quadruplex regulated RNA levels, which would suggest a role in either transcription and/or RNA stability. To perform these studies, we utilized a luciferase reporter construct in which the human APP 3’UTR was inserted after the stop codon of the firefly luciferase gene [49]. To gain insight into the role of the G-quadruplex in regulating expression we created a parallel luciferase construct in which we disrupted the 3’UTR G-quadruplex structure by changing the fourth set of guanine repeats to adenines thereby disrupting tetrad formation [40, 48]; this mutant is analogous to the mutant oligonucleotide sequence used in the CD studies above. To test whether the G-quadruplex affected the expression levels of these transcripts, we transfected HEK293 cells with either construct using identical transfection conditions Using qPCR to measure luciferase mRNA levels in these cell populations, we could detect no differences between luciferase mRNA having either the wild type or mutant G-quadruplex (Fig 3A). The APP 3’UTR G-quadruplex therefore does not play any substantial role in regulating the expression at the level of transcription or RNA stability.

### The APP 3’UTR G-quadruplex Negatively Regulates APP Protein Levels

We then investigated if the APP 3’UTR G-quadruplex plays a role in translation. The wild type and mutant luciferase reporter constructs were individually transfected into HEK293 cells. 24 hours post-transfection, we measured the luciferase activity as a proxy for luciferase protein expression levels from the two constructs. Disruption of the G-quadruplex structure significantly increased luciferase activity by 80% (Fig 3B) compared to the intact, wild type, G-quadruplex. These findings were consistent with results obtained in parallel experiments using HeLa cells (data not shown) indicating that they are independent of the cell type. These findings suggest that translation is more efficient from the construct containing the mutant sequence than that containing the wild type G-quadruplex.

**Fig 4:**
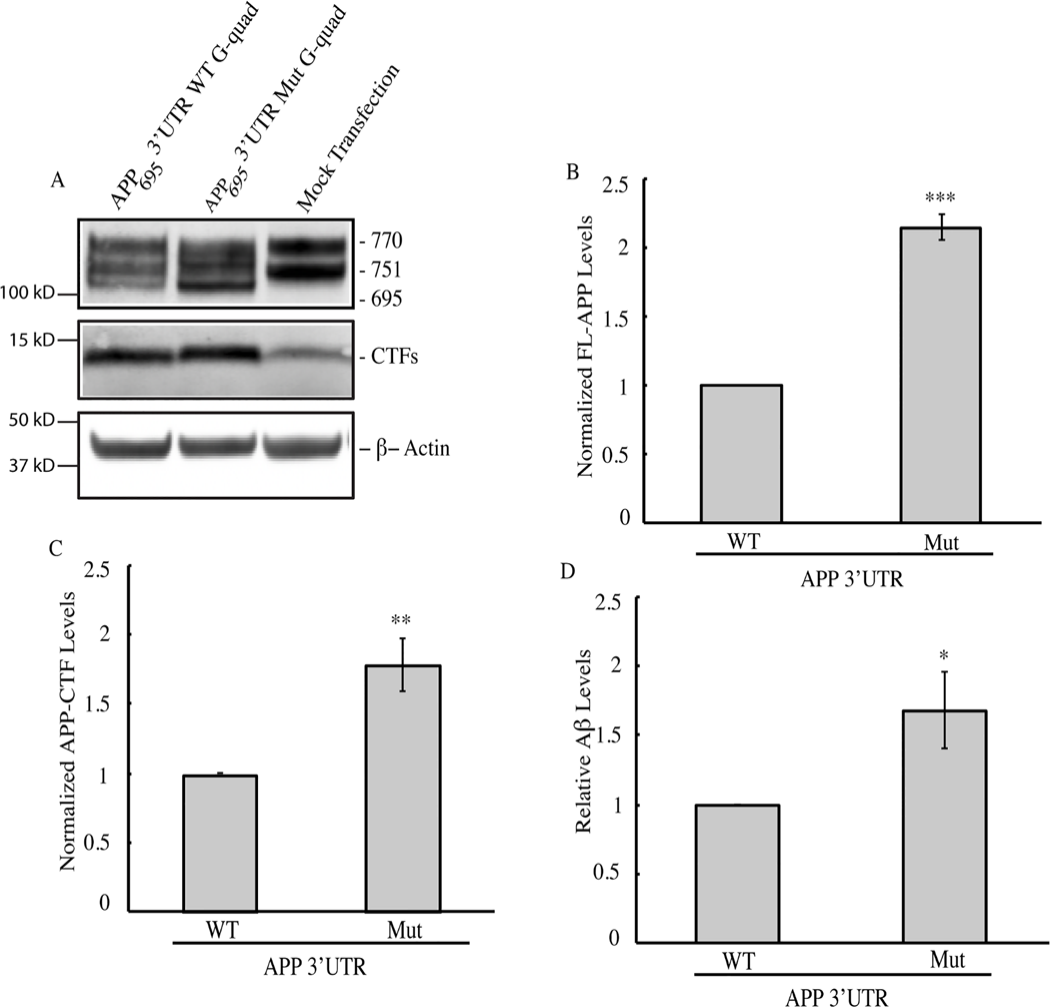
G-quadruplex regulation of APP gene expression. (A) Western blot analysis of cells transfected with reporter constructs containing APP _695_ coding sequence with wild type (G-quad WT) or mutant (G-quad Mut) sequence. Mock Transfection was used to confirm that these constructs were over-expressed in HeLa cells. Antibody C1/6.1 recognizes both full-length APP_695_ and CTF. β-Actin was used as a loading control. (B-C) Quantification of Western blots as in 4A with (B) APP levels normalized to β-Actin and (C) Endogenous CTF intensity values were subtracted from total CTF intensity values to obtain exogenous CTF values which were then normalized to β-Actin values. (D) Aβ ELISA quantification of total Aβ levels from conditioned medium of cells transfected with APP constructs containing the wild type or mutant 3’UTR G-quadruplex.

We next sought to establish a system in which we could measure the effects of this G-quadruplex on APP expression. For these experiments, we used a plasmid encoding the 695 amino acid isoform of human APP (APP_695_) followed by either the wild-type- or G-quadruplex-mutated human APP 3’UTR. These constructs were transiently transfected separately into HeLa cells, which do not express the 695 amino acid isoform of APP [50, 51]. Cell lysates were collected 24 hours post-transfection and subjected to Western blot analysis. Consistent with previous findings [50, 51], we could not detect APP_695_ in untransfected cells (Fig 4A). In contrast, transfected cells expressed readily detectable APP_695_ (Fig 4A). Therefore we can specifically detect exogenous full-length APP_695_ using Western blot analysis.

We then used this system to compare APP expression from cells expressing APP_695_ followed by either the wild type or G-quadruplex-mutated 3’UTR. Mutating the G-quadruplex resulted in an approximate two-fold increase in steady state levels of full-length APP_695_ (Fig 4A and 4B), consistent with the results obtained using the luciferase assay. To gain further insight into APP processing, we measured the abundance of the CTF that was produced from APP_695_ having either the wild type or mutant 3’UTR G-quadruplex. We measured the level of endogenous APP CTF in mock transfected cells. We then measured the levels of exogenous and endogenous APP CTF in cells transfected with the APP695 3’UTR WT G-quad and APP695 3’UTR Mutant G-quad constructs individually. We subtracted the intensity of the APP CTF band of mock transfected cells from that of cells expressing either of the APP constructs to obtain the effect of the 3’UTRs on exogenous APP CTF levels (Fig 4C). This analysis revealed a significant increase in the amount of exogenous APP CTF in the mutant compared to the wild type control. We next investigated whether the increased APP expression led to increased production of Aβ peptides. As expected, ELISA quantification of endogenous and exogenous Aβ indicated that there was a 1.6 fold increase in Aβ levels from cells transfected with APP containing the mutant G-quadruplex sequence compared to the wild type G-quadruplex (Fig 4D). The effects we observe on APP proteolytic cleavage are due to increased APP expression that results from mutating the 3’UTR G-quadruplex. Taken together, these data indicate that the 3’UTR G-quadruplex negatively regulates APP levels.

### Translational Control of APP by G-quadruplex

**Fig 5:**
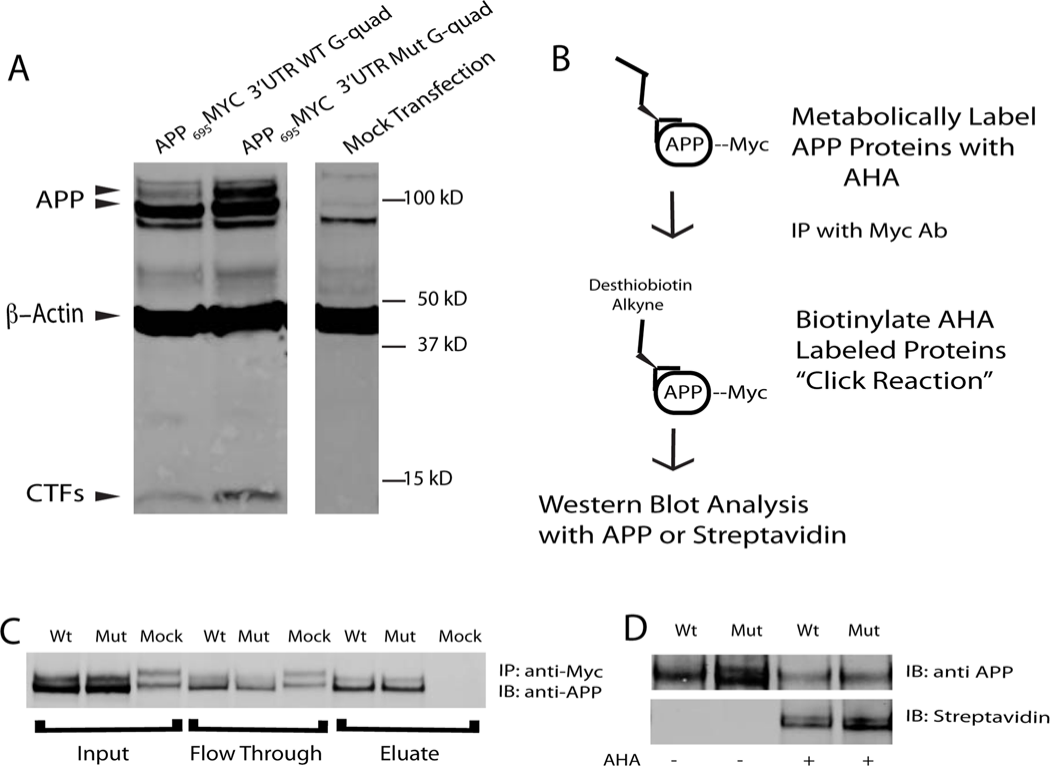
G-quadruplex regulation of APP translation. (A) Western blot analysis of cells transfected with reporter constructs containing APP _695_ coding sequence containing a C-terminal myc tag with either wild type (WT G-quad) or mutant (Mut G-quad) G-quadruplex sequence. Mock transfection was used to confirm that these constructs were over-expressed. Antibody 9B11 (anti-Myc) was used to detect APP-Myc. (B) Schematic representation of methods taken to identify newly synthesized APP (See methods). (C) Western blot analysis for immunoprecipitation demonstrating the successful pull down of Myc-tagged APP constructs (IP: using 9B11 anti myc mAb, IB: C1/6.1 anti APP). (D) Western blot analysis of total APP (top panel, IB: C1/6.1 anti APP) and newly synthesized APP (bottom panel, IB: Streptavidin). Statistical analysis was performed by normalizing the newly synthesized APP/Total APP (Streptavidin/C1/6.1) using a ratio paired t-test using Prism 6.0g for Mac.

Loss of the G-quadruplex leads to an increase in APP protein levels (Fig 4) without affecting the *APP* transcript levels (Fig 3A), suggesting a role for this structure in translational control. To test this prediction, we labeled newly synthesized proteins during a discrete window and asked whether more APP protein was produced from the construct in which the G-quadruplex was mutated. For these experiments, we used HeLa cells that expressed myc-tagged APP constructs followed by either the wild type or G-quadruplex-mutated 3’ UTR (Fig 5A). These cells were metabolically labeled for four hours with L-azidohomoalanine (AHA), a methionine analog that is incorporated into newly synthesized proteins during translation and can be subsequently detected using click chemistry-based approaches. In this approach a desthiobiotin molecule containing an alkyne group forms a covalent bond to the AHA molecule incorporated into newly synthesized proteins (Fig 5B) [52]. Specifically, we immunoprecipitated the exogenous APP using antibodies that recognize myc, biotinylated the AHA-containing APP, and used immunoblotting to determine the extent to which this immunoprecipitated APP had been synthesized during the labeling window (Fig 5C). Using this approach, we determined that the mutant construct resulted in an increase in newly synthesized APP of 19.4% ± 5.3% (mean ± SEM; ratio paired t-test p = 0.0052; n = 6) during the labeling window (Fig 5D). Taken together with the previous findings, these results demonstrate that the 3’UTR G-quadruplex modulates APP protein expression by negatively regulating translation.

## Discussion

In this study, we have analyzed the human APP mRNA sequence for potential regulatory elements and identified a previously uncharacterized G-quadruplex within its 3’UTR. The formation of the intact RNA G-quadruplex was confirmed by CD spectroscopy. Importantly, this structure is stable at physiologic K^+^ concentrations. Using two independent expression constructs, our results demonstrate that this G-quadruplex negatively regulates gene expression in a post-transcriptional manner. In addition to the negative regulation of APP via the 3’UTR G-quadruplex, we further showed that APP overexpression resulting from loss of regulation by the G-quadruplex led to increased Aβ levels. Further studies are needed to elucidate the mechanism by which the APP 3’UTR G-quadruplex regulates APP gene expression.

G-quadruplex-forming sequences can be found throughout mRNAs, including within the 5’UTR, coding regions, and 3’UTR. G-quadruplexes in the 5’UTR have the potential to suppress mRNA translation by blocking initiation factors binding [53] that are required for activating cap dependent translation initiation [54]. G-quadruplexes in the coding regions may have a role in stalling translational elongation [55, 56]. G-quadruplexes in the 3’UTR can be involved in translational repression [57], polyadenylation-dependent mRNA stability [58], and dendritic mRNA targeting [42]. Our data show that the G-quadruplex found in the APP 3’UTR regulates APP gene expression at the translational level.

G-quadruplexes have been found in two other genes whose proteins play crucial roles in APP proteolysis and AD etiology–ADAM10 and BACE1. The α-secretase ADAM10 contains a 5’UTR G-quadruplex that represses translation [59]. BACE1, the β-secretase gene, contains an exonic G-quadruplex that drives BACE alternative splicing. Formation of this G-quadruplex produces a shorter, inactive BACE isoform, while disruption of this G-quadruplex results in the full-length, active BACE isoform which leads to increased Aβ production [60]. The presence of the G-quadruplex in these mRNAs may lead to coordinated regulation and cleavage of APP in response to specific cellular conditions.

Previous work has demonstrated that G-quadruplexes may act as negative regulators of gene expression [61–65] and can exert their regulatory effects by interacting with RNA binding proteins that repress translation [66, 67]. Over three dozen proteins have been reported to bind the APP mRNA [35, 42, 68, 69]. Several of these proteins–including Nucleolin, hnRNP A, Fus, and FMRP–bind to G-quadruplex sequences[67, 70–74]. Whether these proteins interact with the G-quadruplex in the 3’UTR of APP is not known. Moreover, such proteins may interact with the G-quadruplex in concert with other factors; for instance, it has been shown for other mRNAs that RNA binding proteins that interact with G-quadruplexes can require additional protein co-factors to facilitate translational regulation [75, 76]. This remains an important area for future study. Additionally, recent studies have demonstrated that G-quadruplexes may interact with other translational regulators such as microRNAs [76, 77]. Overall, our results show that dysregulated APP translation due to disruption of the G-quadruplex in the 3’UTR lead to elevated levels of APP. Since increased APP levels can lead to AD, it will be of interest to determine whether AD cases arise from dysregulated APP expression due to mutations that disrupt the APP 3’UTR G-quadruplex and/or in the RNA binding proteins that interact with this sequence.
